# PET Molecular Targets and Near-Infrared Fluorescence Imaging of Atherosclerosis

**DOI:** 10.1007/s11886-018-0953-3

**Published:** 2018-02-12

**Authors:** Csilla Celeng, Bart de Keizer, Béla Merkely, Pim de Jong, Tim Leiner, Richard A. P. Takx

**Affiliations:** 1 Department of Radiology and Nuclear Medicine, University Medical Center Utrecht, Heidelberglaan 100, 3584 CX Utrecht, The Netherlands; 2 Heart and Vascular Center, Semmelweis University, Gaál József street 9, Budapest, 1122 Hungary

**Keywords:** Atherosclerosis, Inflammation, Plaque, NIRF, PET

## Abstract

**Purpose of Review:**

With this review, we aim to summarize the role of positron emission tomography (PET) and near-infrared fluorescence imaging (NIRF) in the detection of atherosclerosis.

**Recent Findings:**

^18^F-FDG is an established measure of increased macrophage activity. However, due to its low specificity, new radiotracers have emerged for more specific detection of vascular inflammation and other high-risk plaque features such as microcalcification and neovascularization. Novel NIRF probes are engineered to sense endothelial damage as an early sign of plaque erosion as well as oxidized low-density lipoprotein (oxLDL) as a prime target for atherosclerosis. Integrated NIRF/OCT (optical coherence tomography) catheters enable to detect stent-associated microthrombi.

**Summary:**

Novel radiotracers can improve specificity of PET for imaging atherosclerosis. Advanced NIRF probes show promise for future application in human. Intravascular NIRF might play a prominent role in the detection of stent-induced vascular injury.

## Introduction

Despite all prevention efforts, cardiovascular disease remains one of the leading global causes of death. In 2015, over 7 million deaths worldwide were attributable to the disease [1], which number is expected to rise to more than 23.6 million by 2030 [2]. Cardiovascular diseases encompass several pathological conditions such as coronary heart disease, stroke, and valvular diseases, which are commonly associated with the presence of atherosclerosis. Due to the destructive nature of atherosclerosis, advanced diagnostic imaging techniques have emerged for the detection and characterization of the condition.

Atherosclerosis is the result of a complex process of arterial wall thickening due to immune responses triggered by inherent genetic vulnerabilities and cardiovascular clinical risk factors. Atherosclerosis is most likely initiated by the damage of endothelial cells due to flow disturbances, which lead to over-expression of vascular cell adhesion molecule-1 (VCAM-1), which provokes recruitment of monocytes and T lymphocytes [3]. Monocytes infiltrate the intima and differentiate into macrophages, which becomes filled with lipids and transform to foam cells [4]. Persistent arterial inflammation leads to the proliferation of smooth muscle cells, which in normal circumstances are responsible for healing and repair of arterial injury [5]. Apoptosis of macrophages and smooth muscle cells contribute to plaque instability by promoting the development of a necrotic core [6], which is associated with an increased risk for plaque rupture [7]. Macrophages play a conductor role in the cellular orchestra of atherosclerosis and are therefore attractive targets for imaging.

Positron emission tomography (PET) is a non-invasive diagnostic imaging tool mainly used for cancer imaging, which also allows for the detection of active arterial inflammation. The usefulness of PET for vascular imaging has been successfully demonstrated in multiple studies, including cardiovascular drug trials where PET served as a proxy end point. PET signal correlates with macrophage density in carotid artery plaques [8], inflammatory biomarkers such as C-reactive protein [9] and also with cardiovascular risk factors and the Framingham risk score [10, 11]. The fusion of PET with computed tomography (CT) enables detailed visualization of both functional and anatomical alterations in the atherosclerotic milieu, thus offering incremental prognostic information over PET alone.

Near-infrared fluorescence imaging (NIRF) is another widely investigated technique but as yet it has only been validated to a very limited extent in humans. NIRF uses fluorescent molecular structures (fluorophores) which are capable of biding to various molecular targets such as VCAM-1 molecules [12], oxidized LDL [13••], and smooth muscle cells [14•] but most preferably they connect to macrophage expressed matrix-metalloproteases (MMPs) [15] and cathepsins (cysteine proteases), which initiate the degradation of elastin a structural component of the arterial wall. Besides their elastolytic activity, cathepsins were shown to degrade apolipoprotein B into lipid droplets hence they might play a pivotal role in the development of the lipid-rich necrotic core [16]. Intravascular NIRF uses a specific catheter for sensing intraarterial signs of atherosclerosis. The combination of NIRF catheter with high-resolution imaging techniques such as optical coherence tomography (OCT) or optical frequency domain imaging (OFDI) provides detailed functional and morphological information.

In the past decades, embedding anti-atherosclerotic medication into the medical regime of “vulnerable patients” has reshaped the course of the disease and the concept of vulnerable plaque-related thrombosis is now shifting towards plaque erosion initiated acute coronary syndrome. The development of state-of-the-art imaging techniques, which beyond the morphological signs of atherosclerosis are also able to detect changes in molecular activity, is of utmost importance. The inherent properties of PET and NIRF could fulfill these criteria; thus, in the future further refinement as well as increased use of these promising imaging methods is expected.

## PET Imaging of Atherosclerosis

### Technical Aspects

PET is a non-invasive imaging method, which can detect the activity of physiological and pathological processes in vivo. PET measures annihilation radiation, which occurs during radioactive decay of radiopharmaceutical tracers labeled with positron emitting radionuclides such as ^11^C, ^13^N, ^15^O, and ^18^F (Table 1). The emitted positron annihilates with an electron, which leads to the release of two high-energy (511 keV) photons [17]. Most PET scanners use scintillation detectors to identify the high-energy photons. The interaction of high-energy photons with the scintillation crystals creates tens of thousands of visible “scintillation” photons. These photons are captured by a photomultiplier tube, in which they are accelerated and amplified. Interaction of annihilation photons in the human tissue (Compton scatter) reduces their energy; thus, attenuation of the signal is a major determinant of the image quality of PET, which requires attenuation correction.

**Table 1:** Technical aspects and molecular imaging targets of PET and NIRF

Imaging modality	PET	NIRF
Operating articles	- Positron emitting isotopes (^11^C, ^13^N, ^15^O, ^18^F) - Isotope-labeled radiotracers (glucose, water, ammonia)	- Fluorophores - Proteins, peptides small organic compounds synthetic oligomers and polymers multi-component systems
Manner of Operation	Annihilation of the emitted positron with nearby electrons **↓** radioactive decay	Absorption of light energy of specific wavelength (700–900 nm) and re-emission of photons at a longer wavelength **↓** fluorescence
Emission	Single event: 2 high-energy photons (511 keV)	Cyclic event: 10^9^ photons/s (1.91–1.38 eV)
Event localization	Scintillation detector and photomultiplier tube	CCD camera
Attenuation in the body	Compton scatter: annihilation photons are attenuated depending on the density on the tissue (the more dense the more attenuated)	Deep tissue penetration, diffuse propagation
Quantification of molecular activity	- SUV; signal intensity of a voxel within the region of interest - TBR; arterial wall SUV divided by venous blood SUV - BSV; arterial wall activity subtracted by venous blood activity	- Wavelength in nanometers
Disadvantage	- Strong attenuation in the body, requires attenuation correction - Radiation exposure	- Autofluorescence - Intravascular NIRF is an invasive procedure
Imaging agents and targets	- ^18^F-FDG PET-macrophages - ^18^F-NaF PET-calcification - ^68^Ga-DOTATATE-SSTR2 receptors (macrophages) - ^68^Ga-PENTAXIFOR-CXCR4 receptors (macrophages)	- ProSense 680 and 750-cathepsins B, L, S, and K - MMPSense 680 and GelSense 680-MMP activity - OsteoSense 680-calcium deposition - CLIO-CyAm7 USPIO nanoparticle- macrophages, smooth muscle cells, endothelial cells, thrombosed plaques - LO1-750-oxidized LDL - ICG macrophages and foam cells

*SUV* standardized uptake value, *TBR* tissue-to-background ratio, *BSV* blood subtracted value, *USPIO* ultrasmall superparamagnetic iron oxide, *ICG* indocyanine green

Due to its non-invasive nature and the ability to provide information on biological function PET has become a promising imaging method for the visualization of atherosclerotic processes. Activity of an atherosclerotic plaque is characterized by the accumulation of a given radiotracer. Uptake of the radiotracer can be quantified by its standardized uptake value (SUV), which represents the signal intensity of a voxel within the region of interest. Alternatively, target to background ratio (TBR, arterial wall SUV divided by venous blood SUV) or more recently blood subtracted value (BSV, arterial wall activity subtracted by venous blood activity) have been used for the quantification of arterial inflammation [18, 19]. Hybrid imaging with PET/CT and PET/magnetic resonance (MR) allows for accurate co-registration of metabolic processes to specific anatomic locations.

## PET Molecular Targets of Atherosclerosis

### ^18^F-FDG PET

**Fig. 1:**
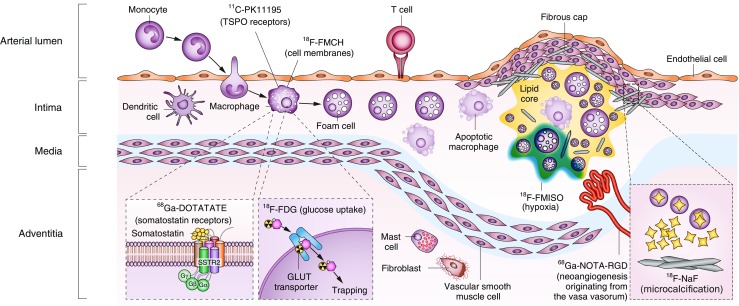
Potential targets for radiotracers in PET imaging of atherosclerosis. Inflammation and underlying pathological mechanisms within high-risk plaques can be detected in vivo by using specific PET tracers. ^18^F-FDG is the most widely investigated and validated PET tracer, which is internalized by macrophages and accumulates proportional to their metabolic activity. The signal of ^18^F-FDG might however be influenced by other factors such as local hypoxia or uptake by cells other than macrophages. Novel PET tracers including ^68^Ga-DOTATATE, ^1^C-PK11195, and ^18^F-FMCH might be more specific for activated macrophages than ^18^F-FDG. Other pathological processes including hypoxia, microcalcification, and neoangiogenesis also contribute to the evolution of vulnerable plaque. These processes can be potentially identified with other novel traces such as ^18^FMISO, ^68^Ga-NOTA-RGD, and ^18^F-NaF. DOTATATE, [1,4,7,10-tetraazacyclododecane-*N*,*N*′,*N*″,*N*‴-tetraacetic acid]-D-Phe^1^,Tyr^3^-octreotate; FDG, fluorodeoxyglucose; FMCH, fluoromethylcholine; FMISO, fluoromisonadazole; GLUT, solute carrier family 2, facilitated glucose transporter member; NaF, sodium fluoride; NOTA-RGD, 1,4,7-triazacyclononane-1,4,7-triacetic acid-Arg-Gly-Asp; SSTR2, somatostatin receptor type 2; TSPO, translocator protein. (Reprinted with permission from Macmillan Publishers Ltd: Nat Rev Cardiol [21], © 2014)

^18^F-FDG is a glucose analogue, which has been linked with macrophage activity (Fig. 1) [20, 21]. Nevertheless, evaluating the coronaries with ^18^F-FDG PET is still challenging, owing to their small size and constant motion. Due to limited spatial resolution (≈ 5 mm) of PET scanners, the measured arterial activity is affected by signal loss to surrounding tissue (spill out) and signal added from neighboring structures (spill in, mainly due to blood activity) [22••]. Background myocardial FDG uptake can be suppressed using a high-fat, low-carbohydrate diet [23]. The ^18^F-FDG circulation time has to be long enough to allow for sufficient FDG accumulation in areas of interest compared to background levels; nevertheless, it has to be as short as possible to allow for efficient workflow and patient comfort. In oncology, a 60-min time slot is commonly used, while in vascular imaging 180 min provides improved quantification [24•]. ^18^F-FDG provides reproducible measures and can be also used to evaluate the effectiveness of anti-atherosclerotic therapies [25, 26]. For example, ^18^F-FDG PET can differentiate the effect of high- vs. low-dose statins on the degree of atherosclerotic inflammation [26].

### ^18^F-NaF PET

Calcification is a hallmark feature of atherosclerosis and CT is widely used to detect macroscopic calcium in the coronary artery tree (i.e., coronary artery calcium/Agatston score), though its triggers remain matter of debate. ^18^F-sodium fluoride (^18^F-NaF) PET has been used as a bone tracer. At the molecular level, fluoride ions interact with hydroxyapatite by ion exchange with hydroxyl groups [27] and uptake of ^18^F-NaF is linked with osteogenic activity. In the context of atherosclerotic plaque imaging, ^18^F-NaF has been used as an in vivo marker of active calcification [28, 29]. ^18^F-NaF is thus capable of detecting early stages of atherosclerosis, namely dedifferentiation of smooth muscle cells resulting in neointimal (micro)calcification (Fig. 1) [21]. Moreover, Dweck et al. [28] observed increased ^18^F-NaF activity only in the culprit lesion, which is thought to reflect subclinical plaque rupture.

### ^68^Ga-DOTATATE PET

^68^Ga-DOTATATE is a novel PET tracer, which has been mainly applied for the detection of neuroendocrine neoplasms, which express somatostatin receptors [30]. ^68^Ga-DOTATATE has high specificity binding affinity for activated macrophages through the somatostatin subtype-2 receptor (SSTR2) [21, 31] (Fig. 1), as such it can be superior to FDG. In mice, ^68^Ga-DOTATATE uptake co-localized with macrophage-rich plaques on immunohistochemical staining [32]. Recently, Tarkin et al. [33] evaluated ^68^Ga-DOTATATE in 42 patients with atherosclerosis. They demonstrated that SSTR2 gene expression is specific for activated proinflammatory macrophages in atherosclerosis and thus ^68^Ga-DOTATATE was capable of identifying culprit vs. non-culprit lesions in patients with acute coronary syndrome. Somatostatin receptors can be also imaged with ^68^Ga-DOTANOC, which besides SSTR2 can bind to SSTR3 and SSTR5 [30]. Despite the coverage of other somatostatin receptor types, ^68^Ga-DOTANOC shows lower signal intensity compared to ^68^Ga-DOTATATE [34].

### ^68^Ga-PENTAXIFOR PET

^68^Ga-PENTAXIFOR has been recently introduced as a PET imaging agent in patients with lymphoproliferative disease [35]. ^68^Ga-PENTAXIFOR shows high affinity to CXCR4 receptors [36], which are expressed by various inflammatory cells including macrophages/monocytes and smooth muscle cells. Targeted imaging with ^68^Ga-PENTAXIFOR was able to identify regional upregulation of CXCR4 receptors in infarcted myocardium of mice and human as well [37••]. In a recent in-human study, ^68^Ga-PENTAXIFOR showed significant association with calcified plaque burden and other cardiovascular risk factors [38••]. Due to its high specificity in the near future, the incorporation of ^68^Ga-PENTAXIFOR to preclinical and clinical studies focusing on atherosclerosis is expected.

## NIRF Imaging of Atherosclerosis

### Technical Aspects

In the past two decades owing to its high sensitivity and resolution as well as absence of radiation, NIRF has emerged as a promising imaging modality for the visualization of atherosclerosis. NIRF is based on fluorescence optical imaging method that uses excitation light from the near-infrared spectrum (700–900 nm) to stimulate fluorescent molecules (fluorophores, contrast agents for NIRF) from ground state (S_0_) to an excited (S_1_, S_2_) state [39]. Relaxation of this excited state to a lower energetic state results in the emission of fluorescence light at longer wavelength. After reaching the ground state, the fluorophore is again available for a new excitation. This highly repetitive action leads to an emission of 10^9^ photons per second per molecule. Inherent properties of near-infrared light such as low absorption and high scattering characteristics allow for deep tissue penetration (to several centimeters) and diffuse expansion [40]. In addition, excitation with near-infrared light in the region of > 750 nm considerably reduces undesired tissue autofluorescence [41] and by improving signal-to-background ratio makes NIRF a highly sensitive imaging tool.

The two most common approaches to detect fluorophores using near-infrared light in deep tissues are fluorescence reflectance imaging (FRI) and fluorescence-mediated molecular tomography (FMT). FRI consists of a laser or a white light source, which excites a fluorescent structure that emits light with different spectral properties and which is eventually captured by a CCD camera. Multi-channel FRI allows for simultaneous detection of different fluorochromes in multiple targets by using suitable filters in front of the CCD camera, which can selectively obtain images with different spectra [42]. FRI is most commonly applied for the visualization of cathepsin B [43], cathepsin K [44], and MMP activity [43].

FMT is the second approach to identify fluorescent contrast agents. It enables isotopic detection as well as absolute quantification of the given fluorophore [45]. The principles of FMT are similar to those in FRI, however, with more profound data collection: generally light from a laser diode is directed through optical fibers to the “optical bore” that surrounds the body of the animal and serves as a CT or MR scanner during the examination. Detection fibers collect the emitted photons and direct them onto CCD camera. FMT can be combined with high-resolution imaging techniques such as CT or MR in order to refine anatomical features [46, 47]. Besides cathepsin B [48], FMT is able to visualize MMP activity [49] as well as fluorescence autoantibodies [13••].

## NIRF Molecular Targets of Atherosclerosis

Increasing knowledge of the pathogenesis of atherosclerosis allows for the identification of novel molecular and structural imaging targets. NIRF molecular imaging of atherosclerotic mechanisms involves the administration of near-infrared fluorophores, which aim to detect and quantify high-risk features of atherosclerosis such as cathepsins S, K, B, L, and F, which are most commonly expressed by macrophages and smooth muscle cells in atherosclerotic plaques [16–18]. One of the most widely investigated NIRF imaging agent in animal studies is ProSense (680 and 750), a copolymer-based smart probe, which is optically silent at baseline (unactivated) and becomes highly fluorescent (activated) after cathepsins B, L, or S protease-mediated cleavage. Using the FRI technique, the cathepsin-activated contrast dye showed strong signal enhancement in macrophage-rich atherosclerotic lesions at the level of the aortic valves in hypercholesterolemic apolipoprotein E-deficient (apoE−/− mice) [43]. By linking a specific cathepsin K (CatK)-sensitive substrate to the copolymer, this NIRF contrast agent is rather cleaved by CatK instead of CatB [44]. Imaging of CatK is of importance as it preferentially co-localizes in macrophages [44] and in vulnerable areas of atherosclerotic lesions, such as the thin fibrous cap, plaque shoulders but it was also detected in ruptured plaques indicating its potential plaque-destabilizing role [44, 50]. Besides cathepsins elevated MMP activity was demonstrated to be strongly associated with unstable atherosclerotic plaques [51, 52]. Gelatinases (such as GelSense 680 or MMPSense 680) are metalloproteinase activatable florescent imaging agents, which demonstrate increased NIRF signal after MMP-mediated activation predominantly released by macrophages rather than smooth muscle or endothelial cells [43, 49]. NIRF imaging of gelatin zymography was also able to differentiate hot and cold spots (areas with relatively high and low signal intensity) across the plaque surface which might indicate the presence of lesion instability [15]. Monitoring vascular response after stent implantation is also feasible with MMP activated fluorochromes. FMT analysis showed significantly increased MMP activity in stented aortas of apoE^−/−^ mice compared to wild-type (WT) mice [53]. These findings were also confirmed by real-time PCR, which revealed significantly more transcripts encoding for MMP-2, MMP-9, and MMP-13 in apoE^−/−^ mice than those in WT mice. Other atherosclerotic processes such as increased osteoblastic activity as an early precursor of calcium deposition can be also targeted by fluorescent bisphosphonate imaging agents (OsteoSense 680) [43].

As the focus from thin fibrous cap rupture shifts towards superficial erosion related plaque thrombosis [54••], novel imaging targets emerge for the visualization of the associated pathological mechanisms. Endothelial cell damage induced by shear stress leads to the development of impaired endothelial permeability and may indicate future presentation of superficial erosion [55]. To address this hypothesis a CLIO-CyAm7 a NIRF-derivatized ultrasmall superparamagnetic iron oxide (USPIO) nanoparticle was engineered and applied in rabbits on high-cholesterol diet [14•]. CLIO-CyAm7 accumulated in atherosclerotic plaques, primarily in the superficial intima within macrophages, smooth muscle cells, endothelial cells, and thrombosed lesions. Heterogeneous distribution of CLIO-CyAm7 across the plaque surface as well as its deposition in deeper areas with neovascularization indicated regional alterations in endothelial permeability. CLIO-CyAm7 USPIO nanoparticle therefore might be useful for the detection of high-risk atheroma as well as early signs of superficial erosion.

State-of-the are NIRF dye-labeled monoclonal autoantibodies aim to identify and quantify oxidized low-density lipoprotein (oxLDL) as prime target of atherosclerosis. Specifically, in a recent study LO1 monoclonal autoantibody (which is able to react with oxLDL) was labeled with NIRF dye (LO1-750) and its uptake was analyzed in high fed (HF) atherosclerotic LDLr^−/−^ mice and WT mice [13••] on FMT-CT images. In addition, the signal activity of LO1-750 was compared to MMP-activatable (MMPSense-645-FAST) fluorescent probe. After the injection of LO1-750 into LDLr^−/−^ mice, a clear accumulation was observed in the aortic arch and its branches. Quantitative analysis of LO1-750 revealed a significantly higher uptake by LDLr^−/−^ mice compared to WT mice (25.3 ± 4.6 vs. 1.3 ± 0.9 pmol; *P* < 0.005). LO-750 in LDLr^−/−^ to WT mice gave superior signal ratio comparing to MMPSense (19.3 vs. 2.8, *P* = 0.03). Furthermore, a generated partially humanized chimeric LO1-Fab-Cys-750 construct localized similarly to the parent antibody in mice atherosclerotic lesion showing potential for future application in humans [13••].

The use of NIRF imaging for the visualization of atherosclerosis is mainly limited due to lack of clinically approved fluorophores for human use. To date, indocyanine green (ICG) is the only US Food and Drug Administration-approved contrast agent that can be employed for the evaluation of hepatic function [56], cardiac output [57], and retinal angiography [58] on the basis of its dark green color. ICG is an amphiphilic contrast dye (it has both hydrophilic and lipophilic properties) and is able to interact rapidly with HDL and LDL [59]. Furthermore, it showed reliable detection of inflammatory alterations in arthritis [60]. Owing to these characteristics in the past decade, the capability of ICG to identify inflamed atherosclerotic lesions was intensively investigated. A study by Vinegoni et al. [61] demonstrated that ICG primarily accumulates in lipid and macrophage-rich areas of atherosclerotic plaques in rabbits. In the in vitro part of the study, they also showed that through direct binding to LDL or albumin human macrophages and foam cells are also able to internalize ICG.

With the use of a combined OCT-NIRF technique, the same group conducted the first-in-human trial, which aimed to visualize atherosclerotic lesions in patients prior to carotid endarterectomy with the administration of ICG [62•]. OCT-NIRF of the resected carotid portions detected evident ICG signals in all patients injected with ICG with higher signal intensity of extensively stenotic vessels.

## NIRF Intravascular Imaging of Atherosclerosis

In 2008, Farouc et al. [63] developed a NIRF catheter-based imaging technique to detect intravascular sings of atherosclerosis in vivo. The catheter was designed to sense fluorescence signal of an area of ≈ 40 μm diameter with a distance of ≈ 2 mm from the catheter, however, without rotation and pullback function thus was operating in a one-dimensional manner. The developed 90°-sense catheter was able to detect NIRF signals attributable to cysteine protease, specifically cathepsin B activity in rabbit iliac arteries. The same group later addressed the limitations of this catheter and in 2011 developed a two-dimensional rotational NIRF catheter, with automatic pullback function in order to provide new insights to arterial inflammation and stent healing process in vivo [64]. The 2D intravascular NIRF catheter was able to provide real-time images of cathepsin B activity as well as of elevated signal levels at the distal edges of the implanted stents, which might suggest that in the injured vessels the damage presents at sharp transition zones. The capability of NIRF for the detection of stent-induced vascular injury might elucidate the confusing data over bare metal stents vs. drug eluting stent-associated events [65–67].

**Fig. 2:**
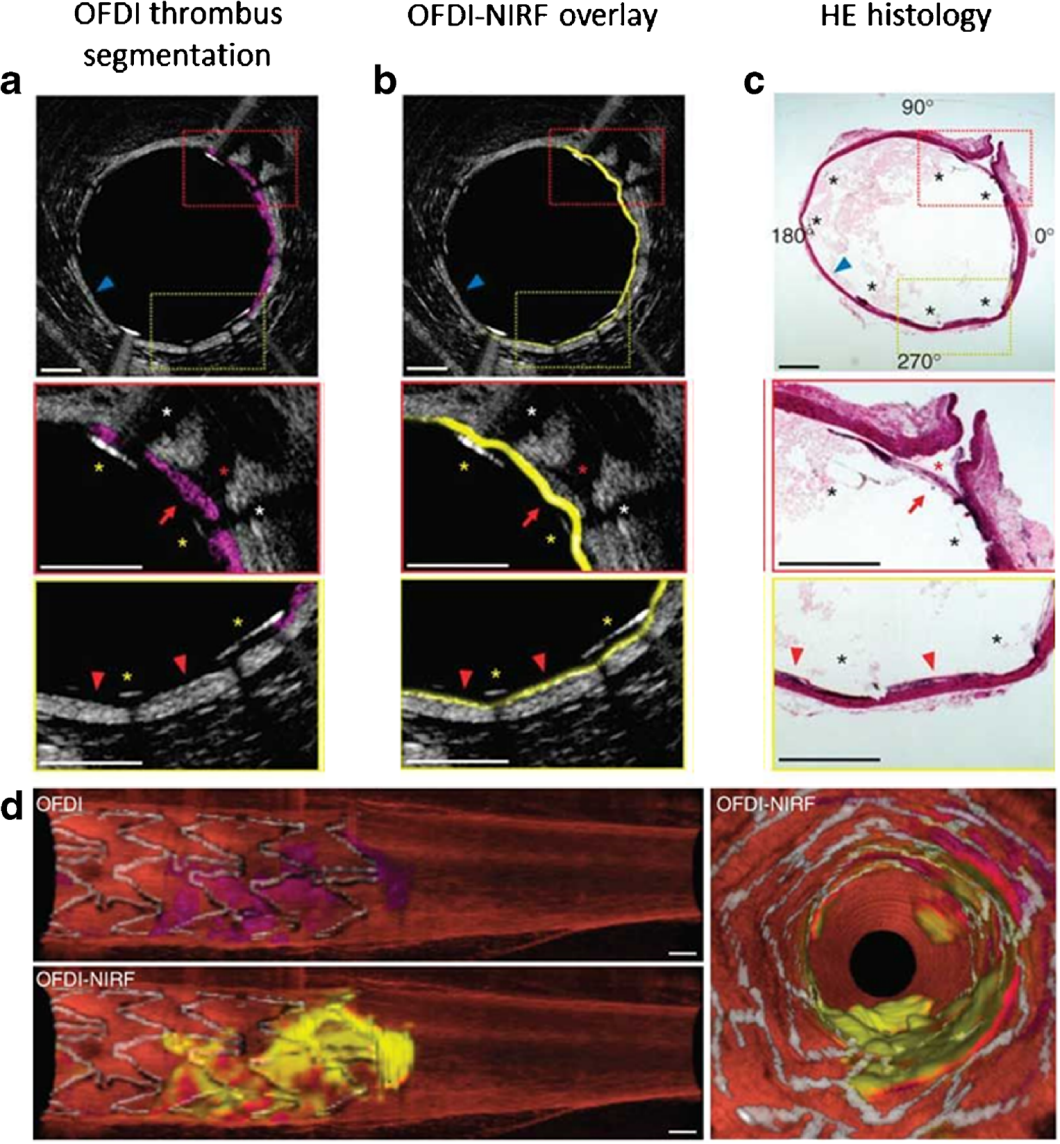
Integrated OFDI-NIRF images of a rabbit iliac artery with an implanted NIRF fibrin-coated stent, attained in vivo*.* **a** OFDI (gray scale) with thrombus segmentation (*purple*). **b** OFDI (gray scale)-NIRF (yellow scale) overlaid images. **c** Corresponding HE histology images. Middle rows demonstrate zoomed images of the thrombus (red arrow), stent struts (yellow asterisks, black asterisks in HE images), and their shadow (white asterisk). Bottom rows show zoomed images of an area (red arrowheads), which was thrombus negative according to OFDI; however, NIRF detected a weaker fluorescence signal, which was also confirmed by histology. **d** Three-dimensional image of a stented right iliac artery of a living rabbit. Structural components were segmented and color-coded in OFDI images for clear visualization. Red: artery wall; white: stent; purple: thrombus; yellow: near-infrared fluorescent fibrin. Scale bars, 500 μm. (Reprinted with permission from Macmillan Publishers Ltd: Nat Med [69], © 2011)

NIRF imaging was also combined with high-resolution imaging techniques such as OFDI, which owing to its high-resolution and high frame-rate is able to visualize the detailed three-dimensional microstructure of the arterial wall [68]. An engineered hybrid NIRF-OCDI catheter allowed for concomitant assessment of molecular and microstructural characteristics of high-risk plaques and stent thrombosis in rabbits in vivo (Fig. 2) [69]. One of the limitations of the dual-modality NIRF-OCDI imaging is the manual adjustment of the detected NIRF map with the corresponding OFDI vessel wall position, which is a time-consuming process. To overcome this obstacle, a fully automated algorithm was developed and validated in previously manually segmented rabbit and human artery images [70]. Results showed high similarity correlation between the manual and fully automatic method as well as greatly reduced processing time (44 ms vs. 1 h or more), suggesting that more frequent interpretation of NIRF-OCDI in the future is expected.

Previous studies by Lee et al. [71•] demonstrated the feasibility of real-time structural/molecular imaging by combining OCT data with NIRF. The integrated OCT-NIRF catheter was able to simultaneously co-localize the morphological and pathological alterations of rabbit atherosclerotic plaques targeted with ICG exogenous contrast dye [71•]. One step further, the same group also showed the capability of the integrated OCT-NIRF catheter to identify high-risk plaques and stent-related inflammation in beating swine coronary arteries [72].

Recently, red excited (633 nm) near-infrared autofluorescence (NIRAF) is another profoundly investigated imaging method [73], as it does not require the administration of exogenous contrast agent, which property might facilitate its early adoption in the clinical routine. The incorporation of NIRAF with OCT was able to provide high-quality imaging data of coronary atherosclerotic lesions in patients undergoing percutaneous coronary angiography [74•]. An increased NIRAF signal was significantly associated with thin-cap fibroatheroma and plaque rupture defined by OCT.

## Conclusions

Current approach to personalized medicine resulted in advanced imaging tools for the evaluation of atherosclerosis. These new imaging techniques will further enhance our understanding of the disease mechanisms. PET imaging allows for the direct visualization of metabolic processes, including plaque inflammation, bone formation, as well as macrophage activity, which is already widely studied in humans. Besides the tracers discussed in this review, novel ^18^F-labeling methods will enable the synthesis of specifically labeled PET tracers, thus enabling more specific assessment of in vivo pharmacokinetics.

NIRF molecular imaging agents are designed to reveal premature signs of atherosclerosis on a molecular level and have potential to identify individuals who might benefit from early preventive therapy. NIRF is however still in investigational phase and its use in clinical practice will require long-term clinical trials. It is expected that in the near future, state-of-the-art fluorophores with desirable architecture such as high solubility and photon emission will be validated in humans. Intravascular NIRF molecular imaging especially OFDI-NIRF or OCT-NIRF platforms are capable to provide real-time microscopy-resolution images of molecular as well as structural changes of the arterial wall. Beyond identifying high-risk features of atherosclerosis, NIRF intravascular molecular imaging is also able to assess response to implanted stents including potential thrombotic apposition, therefore, might play a prominent role in adjustment of the applied medical regimens such as antiplatelet and statin therapy. In addition, the use of automatic algorithms for image processing can greatly contribute to its faster clinical utilization.

## Abbreviations

BMS: Bare metal stent
BSV: Blood subtracted value
Cat: Cathepsin
CT: Computed tomography
DES: Drug eluting stent
FMT: Fluorescence-mediated molecular tomography
FRI: Fluorescence reflectance imaging
FDG: Fluorodeoxyglucose
ICG: Indocyanine green
MMP: Matrix-metalloprotease
MR: Magnetic resonance
NIRAF: Near-infrared autofluorescence
NIRF: Near-infrared fluorescence imaging
OCT: Optical coherence tomography
OFDI: Optical frequency domain imaging
OxLDL: Oxidized low-density lipoprotein
PET: Positron emission tomography
SSTR2: Somatostatin subtype-2 receptor
SUV: Standardized uptake value
TBR: Target to background ratio
VCAM-1: Vascular cell adhesion molecule-1
USPIO: Ultrasmall superparamagnetic iron oxide
